# State-of-the-Art Exercise Concepts for Lumbopelvic and Spinal Muscles – Transferability to Microgravity

**DOI:** 10.3389/fphys.2019.00837

**Published:** 2019-07-04

**Authors:** Julie Hides, Paul Hodges, Gunda Lambrecht

**Affiliations:** ^1^ School of Allied Health Sciences, Griffith University, Nathan, QLD, Australia; ^2^ Mater Back Stability Research Clinic, Mater Health, South Brisbane, QLD, Australia; ^3^ School of Health and Rehabilitation Sciences, NHMRC Centre of Clinical Research Excellence on Spinal Pain, Injury and Health, The University of Queensland, Brisbane, QLD, Australia; ^4^ European Space Agency Space-Medicine Office, European Astronaut Centre, Cologne, Germany; ^5^ Germany Praxis fur Physiotherapie und Osteopathische Techniken, Siegburg, Germany

**Keywords:** counter-measures, trunk muscles, anti-gravity, space flight, back pain

## Abstract

Low back pain (LBP) is the leading cause of disability worldwide. Over the last three decades, changes to key recommendations in clinical practice guidelines for management of LBP have placed greater emphasis on self-management and utilization of exercise programs targeting improvements in function. Recommendations have also suggested that physical treatments for persistent LBP should be tailored to the individual. This mini review will draw parallels between changes, which occur to the neuromuscular system in microgravity and conditions such as LBP which occur on Earth. Prolonged exposure to microgravity is associated with both LBP and muscle atrophy of the intrinsic muscles of the spine, including the lumbar multifidus. The finding of atrophy of spinal muscles has also commonly been reported in terrestrial LBP sufferers. Studying astronauts provides a unique perspective and valuable model for testing the effectiveness of exercise interventions, which have been developed on Earth. One such approach is motor control training, which is a broad term that can include all the sensory and motor aspects of spinal motor function. There is evidence to support the use of this exercise approach, but unlike changes seen in muscles of LBP sufferers on Earth, the changes induced by exposure to microgravity are rapid, and are relatively consistent in nature. Drawing parallels between changes which occur to the neuromuscular system in the absence of gravity and which exercises best restore size and function could help health professionals tailor improved interventions for terrestrial populations.

## Introduction

Exposure to microgravity induces rapid alterations in multiple physiological systems. This mini review focuses on neuromuscular and sensorimotor systems. One of the primary changes in the neuromuscular system in response to microgravity is skeletal muscle atrophy, which occurs especially in muscles that maintain posture while upright on Earth (anti-gravity or postural muscles) (Chang et al., 2016). Changes are greater in the lower body than the upper body, with muscle losses documented in the muscles spanning the trunk, hip, knee, and ankle (Mulavara et al., 2018). In addition, bone mineral density decreases by 1–2% per month (LeBlanc et al., 2000). Back pain among astronauts has a documented incidence rate of 52 (Kerstman et al., 2012) and 70% (Pool-Goudzwaard et al., 2015). Exercise is the only countermeasure available to mitigate declines in muscle size and function, bone density, and is routinely used in spaceflight to protect health of crews and to effectively manage back pain (Kerstman et al., 2012). The NASA roadmap projects that humans will be sent to Mars by the 2030s. Details concerning how exercise can be optimized to limit atrophy are currently inadequate, and development of countermeasures for long duration missions is required. A recent study reported that lessons learned from development of exercise strategies on Earth could inform programs for long duration missions. In particular, programs developed for people with low back pain (LBP) may have application as inflight exercise countermeasures (Hides et al., 2017). Since the last review was written in 2017, there have been considerable advancements in knowledge in the field of LBP research. For example, the mechanisms underpinning the changes in paraspinal muscles observed in people with LBP are only just beginning to be understood. An increased understanding of these changes may inform improved design and timing of exercise interventions. Despite there being a small amount of data for astronauts, and a large amount of data for people on Earth with LBP, studying astronauts provides valuable data as exposure to microgravity provides a discrete perturbation to the system and produces relatively consistent and predictable responses. On Earth, LBP and the accompanying changes in the paraspinal muscles might take decades to occur and have many contributing factors. With astronauts, it is possible to plan longitudinal studies and conduct measures pre-and post- exposure to the discrete modification of microgravity and evaluate the effectiveness of different interventions. Conducting prospective research trials on Earth is difficult, as it requires assessment of a very large pool of individuals, waiting for LBP to develop, and accounting for the multiple contributing variables. Additional information may be gained from Earth-based analogue studies, such as exercise interventions for people following prolonged bed rest.

## Earth-Based Analogue Studies and Trunk Muscles

Bed rest and dry immersion are models commonly used to simulate microgravity and create a terrestrial model of space flight with the advantage of being able to manipulate research conditions (Parry and Puthucheary, 2015; Ploutz-Snyder et al., 2018; Tomilovskaya et al., 2019). In bed rest, participants follow a strict protocol of lying down in bed at a 6° head down tilt for days to months. In dry immersion, a waterproof elastic fabric is used to immerse subjects into a deep bath up to the neck level in a supine position (Tomilovskaya et al., 2019). These trials are used to understand the implications of muscle disuse/physical inactivity, to simulate the axial unloading experienced by the sensorimotor system in space and in the case of dry immersion, to simulate a lack of support (Reschke et al., 2009; Tomilovskaya et al., 2019).

In the trunk region, muscles that have been studied include the multifidus, lumbar erector spinae, quadratus lumborum, abdominal wall muscles (transversus abdominis, and obliquus internus and externus abdominis) and psoas. Bed rest studies have shown that preferential atrophy of antigravity muscles occurs in response to this stimulus (Bloomfield, 1997), and there is evidence of progressively greater decrements in muscle size over time (Parry and Puthucheary, 2015). In dry immersion, a significant decrease in the transverse stiffness of the back extensor muscles has been noted (Rukavishnikov et al., 2017). This is interpreted to indicate decreased extensor muscle activity and produce a flexed posture as an acute response to transition to weightlessness (Tomilovskaya et al., 2019). In response to bed rest, the rate of muscle atrophy in the lumbo-pelvic region was greatest in the multifidus (L4 and L5 vertebral levels) and the lumbar erector spinae (at L1 and L2) muscles, where both muscles are known to have their greatest cross section area. A 5-day dry immersion study also found decreased cross-sectional area area of the quadratus lumborum, multifidus, and erector spinae muscles at the L4-L5 vertebral level on MRI (Rukavishnikov et al., 2018). Bed rest induced a contrasting increase in size of the abdominal flexor and psoas muscles (Hides et al., 2007; Belavy et al., 2011). Some of the changes induced in prolonged bed rest studies are long lasting in nature. Changes observed in the lumbar multifidus muscles remained evident 90 days following re-ambulation and return to full pre-bed rest levels of activity (Belavy et al., 2008).

In addition to changes in muscle size, bed rest, and dry immersion have been found to affect the passive structures of the spine. This includes increased disc volume, spinal length, and loss of the lower lumbar lordosis (Belavy et al., 2011; Rukavishnikov et al., 2018). Although these Earth-based analogue studies provide valuable insights, some responses may not be observed in microgravity. For example, no changes in lumbar inter-vertebral disc height or disc water content were found on pre- to post- space flight imaging (Chang et al., 2016; Bailey et al., 2018) and the occurrence of LBP on Earth during bed rest is different from LBP in microgravity in relation to pain intensity and duration (Pool-Goudzwaard et al., 2015). Innovations including use of in-flight ultrasound imaging are being evaluated as a way to monitor changes occurring during space flight to understand the mechanisms for these differences (Garcia et al., 2018; Harrison et al., 2018).

## Changes in Trunk Muscles Associated with Low Back Pain and Spinal Conditions

Low back pain is a complex condition with multiple contributors to both the pain and associated disability, including psychological factors, social factors, biophysical factors, comorbidities, and pain-processing mechanisms (Hartvigsen et al., 2018). This mini review will address biophysical factors, namely trunk muscle changes. Changes in trunk muscles have been observed in people with LBP. In those with acute LBP, localized muscle atrophy of the multifidus muscles has been demonstrated (Hides et al., 1996). In subacute LBP, there is adipose accumulation without atrophy (Battie et al., 2012), whereas chronic LBP is characterized by more diffuse atrophy (Hides et al., 2011), fibrosis and fatty infiltration (Zhao et al., 2000). A recent systematic review examined the association between LBP and morphology of paraspinal muscles including the erector spinae, multifidus, psoas, and quadratus lumborum muscles (Ranger et al., 2017). Results showed evidence for a negative association between cross-sectional area (CSA) of the multifidus muscles and LBP (smaller muscle as related to worse LBP). Results were conflicting for the other muscles (Ranger et al., 2017). CSA of the multifidus muscles was predictive of LBP for up to 12 months in men (Ranger et al., 2017) and CSA of the multifidus and erector spinae muscles at the L4 and L5 vertebral levels predicted low back disability (Ranger et al., 2018). Fatty infiltration of paraspinal muscles may result in loss of muscle function and impaired strength (Lang et al., 2010). Although higher levels of MRI defined fat infiltration have been observed in people with LBP (Kjaer et al., 2007; Pezolato et al., 2012), there are conflicting results regarding the relationship between fatty infiltration of the multifidus muscles and LBP (Ranger et al., 2017). The mechanisms underpinning the changes in the paraspinal muscles in people with LBP are complex and time dependent and only beginning to be understood. In the acute phase, animal studies show reduced neural drive to the multifidus muscles (consistent with inhibition) immediately after injury (Hodges et al., 2009). However, this appears to shift to fibrotic, adipose and muscle fiber-type changes (fast-to-slow fiber transformation) in the multifidus muscles mediated by dysregulated inflammatory pathways in the muscle in the subacute period (Hodges et al., 2015), which is thought be related to activity of pro-inflammatory macrophages (James et al., 2018b). In animals, this has been shown following experimental injury to intervertebral disc despite no direct injury to the muscle (Hodges et al., 2009), and after spontaneous intervertebral disc degeneration (James et al., 2018a, 2019). In this latter study, dysregulation of the inflammatory pathways was related to the severity/extent of the disc degeneration and changes were prevented by physical activity (James et al., 2018a). Exercise also prevented the accumulation of fibrosis (James et al., 2019). In addition to these effects, exercise also polarizes macrophages to the anti-inflammatory subtype (Leung et al., 2015) in addition to other effects such as preventing central sensitization (Sluka et al., 2013), as well as effects on muscle fiber types and metabolism. Together, exercise represents a clinical strategy with diverse effects on muscle health, supporting the concept of ‘exercise as medicine’ (Ploutz-Snyder et al., 2018). In the chronic phase, more generalized changes appear consistent with disuse.

Findings of a pro-inflammatory response in the multifidus muscles may also be very relevant for systemic inflammatory conditions affecting the spine such as axial spondyloarthritis (axSpa) and for critically ill patients who are immobilized. Although axSpa is a systemic disease, the initial inflammatory changes occur in the lumbo-pelvic region, and the lumbar paraspinal muscles are therefore a target for any primary or secondary pathological changes. Studies using MRI have demonstrated decreased CSA of the multifidus muscles, as well as changes in composition of the muscles (Akgul et al., 2013; Resorlu et al., 2017). Although exercise is beneficial in diseases such as axSpa, the mechanisms are not known. The effects could be mediated by the complex physiology of cytokines and associated molecular pathways. For instance, cytokines including interleukin 6 (IL-6) are released by muscles on contraction (Ostrowski et al., 2000). IL-6 has both pro- and anti-inflammatory effects and can act in a hormone like manner to produce anti-inflammatory effects, such as increasing IL-10, while decreasing TNF-α (Starkie et al., 2003). On this foundation, it has been proposed that the benefits of exercise on axSpa may be mediated *via* decreasing inflammation (Sveaas et al., 2017). Regarding critically ill patients, immobility also increases the production of pro-inflammatory cytokines and reactive oxygen species with subsequent muscle proteolysis promoting overall muscle loss (Winkelman, 2009; Puthucheary et al., 2010).

## Exercise and Low Back Pain

There are many forms of exercise therapy for low back pain (LBP). Over the last three decades, changes to key recommendations in clinical practice guidelines for management of LBP have placed greater emphasis on self-management and exercise programs targeting functional improvement (Foster et al., 2018). Many approaches for management of LBP focus on modifying motor control, which refers to motor, sensory, and central processes for control of posture and movement (Hides et al., 2019). A common assumption of motor control training (MCT) is that the manner in which an individual loads their spine (e.g. posture, movement, and muscle activation strategies) can contribute onset, persistence, and recovery of symptoms. MCT considers sensory and motor aspects of spine function, and each individual’s management program is tailored to the suboptimal features identified on assessment. The MCT approach aims to identify and modify the suboptimal features of motor control, with integration into function. Although there is limited evidence to suggest that MCT is more effective for LBP than other forms of exercise in the general population (Macedo et al., 2014; Smith et al., 2014; Saragiotto et al., 2016), MCT has been considered to be an important component of post-mission neuromuscular reconditioning of astronauts post spaceflight, especially with respect to regaining postural alignment and axial loading (Evetts et al., 2014).

It has been demonstrated that MCT can remediate changes in trunk muscles associated with LBP, but the effects and design of exercise will depend on the timing, and the underlying mechanisms. In the acute phase, when neural inhibition explains the rapid muscle atrophy, MCT achieved restoration of CSA of the multifidus muscles (Hides et al., 1996) and reduced recurrence of symptoms (Hides et al., 2001) with precise gentle activation. In athletes with more persistent symptoms, MCT decreased pain along with increases in the CSA of the multifidus muscle (Hides et al., 2008). Several studies have shown that in the chronic phase, adequate loading of the muscles is necessary to induce muscle hypertrophy (Danneels et al., 2001; Schoenfeld, 2010). Of note, individuals with LBP who have higher proportions of fatty infiltration into lumbar multifidus (at the L4/5 and L5/S1 vertebral levels) are less likely to respond to exercise therapy. This may suggest that structural changes in muscles are more resistant to change (Hebert et al., 2018), or that further refinement of the exercise design is necessary to optimize effect in this group. Promisingly, there is preliminary evidence that fatty infiltration of the multifidus muscles can be reduced with exercise. A recent study showed that free weight-based resistance training decreased chronic LBP and disability, in conjunction with altered biomechanics of a squat exercise and reduced fatty infiltration of lumbar multifidus and lumbar erector spinae muscles at the L3/4 and L4/5 vertebral levels, but not at L5/S1 (Welch et al., 2015). The L5/S1 vertebral level had higher percentages of fatty infiltration pre-intervention, and the investigators proposed that muscles with a higher percentage of fatty infiltration may be more resilient to change in response to exercise, or alternatively that the loading may have been distributed unevenly with decreased loading on the multifidus muscle in that region.

## Effects of Microgravity on Trunk Muscles

Recent work has investigated the effect of microgravity on active (Chang et al., 2016; Bailey et al., 2018; Burkhart et al., 2018) and passive structures of the spine (Garcia et al., 2018; Harrison et al., 2018). Both the size and composition of the paraspinal muscles and changes in the lumbar lordosis have been examined pre- and post-spaceflight and 6 months on the International Space Station (ISS) in three recent studies (Chang et al., 2016; Bailey et al., 2018; Burkhart et al., 2018). The lumbar spine was shown to flatten by 11%, and the size of the multifidus muscles decreased by 8–9% (at the L3-4 vertebral level) (Bailey et al., 2018). Of note, changes in multifidus CSA correlated with the changes in the lumbar lordosis. With respect to individual paraspinal muscles decreases in CSA (erector spinae: −4.6%, multifidus: −8.4%, quadratus lumborum: −5.9 to −8.8%) and increased intramuscular fatty infiltration of these muscles (and the psoas) have also been observed post-flight (Burkhart et al., 2018). However, CT scanning was conducted at the L1/L2 vertebral levels, so is possible that the changes observed may have been even greater at the lower lumbar levels, which were not measured in this study. Promisingly, results showed that more resistance exercise was associated with less decline in the CSA of the ES and MF muscles (Burkhart et al., 2018). A study employing MRI showed that paraspinal lean muscle mass at the L3-4 vertebral level decreased significantly post mission, but recovery was incomplete 46 days post-flight (Chang et al., 2016). It is currently unknown whether exercise countermeasures will be effective at preventing inflight paraspinal muscle atrophy in long duration missions (Chang et al., 2016).

## Current Exercise Countermeasures on the ISS

Since 2006, the European Space Agency (ESA) has built a multidisciplinary team that is responsible for astronaut preparation, inflight management while on the ISS and reconditioning after return to Earth. A recent clinical commentary has provided a detailed description of the physiotherapy (Lambrecht et al., 2017) and sports science (Petersen et al., 2016) programs. These were developed over nine long-duration missions. There is also work outlining the Russian countermeasure systems for adverse effects of microgravity on long-duration ISS flights (Kozlovskaya et al., 2015). The principles underlying the ESA astronaut program for lumbo-pelvic neuromuscular reconditioning post spaceflight have also been published (Evetts et al., 2014).

During pre-flight training, astronauts are familiarized with the Advanced Resistive Exercise Device (ARED), which is an exercise countermeasure on the ISS. This focuses on optimizing spinal posture during the exercise while on Earth, as maintaining a good spinal position in microgravity can be challenging due to the reduced awareness secondary to lesser proprioceptive feedback in the absence of gravitational load and muscle activation. In flight, astronauts perform 2 h of training each day to mitigate the known negative effects of microgravity on the neuro-musculoskeletal system. A comprehensive program including use of cycle ergometry, treadmill and ARED training is used in an attempt to maintain muscular and cardiovascular endurance, muscle strength, and provide axial loading of skeletal structures (Lambrecht et al., 2017). However, it is important to note that the vertical loading provided by a harness on the ISS treadmill only provides a load of approximately 50 to 70–80% of body weight (Petersen et al., 2016) and that previous work has shown peak forces experienced during walking and running on-orbit are markedly lower than those measured on Earth (Cavanagh et al., 2010). Astronauts are monitored using real-time feedback *via* an audio and video conference link with the ESA physiotherapist and sports scientist, to optimize performance and for safety (Lambrecht et al., 2017).

Longitudinal monitoring of size of trunk muscles in response to exposure to microgravity and reconditioning has shown that exercises performed using the ARED induce changes that differ between trunk muscles/muscle regions. Although the exercise program successfully maintained the size of the multifidus muscles at the L2-L4 vertebral levels, the multifidus muscle at the L5 level was still reduced post exposure to microgravity (Hides et al., 2016). The localized effect and recalcitrance to rehabilitation at this level parallels findings of some Earth based studies. The multifidus muscle at L5/S1 has been shown to be affected more than other vertebral levels in response to de-loading (Hides et al., 2007; Belavy et al., 2011), acute and chronic LBP (Hides et al., 1996, 2011) and in response to exercise interventions (Hides et al., 2008, 2012; Welch et al., 2015). The observation that size of the multifidus muscles at L4 and L5 predicts disability associated with LBP, reinforces the premise that these lower levels require special attention when prescribing exercise (Ranger et al., 2018). The position of the lumbo-sacral junction has been monitored closely in astronaut reconditioning to allow progression to weightlifting and endurance training for astronauts (Petersen et al., 2017). If the astronaut is unable to control their spinal alignment during exercise and function, they are encouraged to exercise with lower load, where optimal postural alignment can be maintained, prior to progression to greater load.

## Implications for Exercise Countermeasures for Future Human Exploration Missions

One of the challenges for space travel beyond the ISS is the development of effective countermeasures for future exploration vehicles that are highly restricted in terms of the allocations for exercise hardware, volume, mass, and power (Ploutz-Snyder et al., 2018). Thus, large devices such as the ARED may not be available. NASA is currently developing small exercise devices that combine aerobic and resistance exercise in a single device (Ploutz-Snyder et al., 2018). When tested on bed-rest participants, 1 h per day preserved muscle, cardiovascular fitness and bone mass. This training time is shorter than daily exercise sessions performed on the ISS (Lambrecht et al., 2017), and the smaller size of the device could potentially be used in volume constrained spaceflight. Lower body negative pressure treadmill exercise has also been implemented on bed-rest participants, with findings suggesting this intervention partially counteracts deconditioning associated with simulated microgravity (Cao et al., 2005). It has been proposed that the proprioceptive system could be targeted by countermeasures given current spaceflight constraints (Layne and Forth, 2008; Yarmanova et al., 2015; Mulavara et al., 2018). Neurophysiological studies indicate that when vestibular information becomes unreliable, supplemental information such as proprioception is up-weighted to maintain control of posture and locomotion (Yates et al., 2000; Carriot et al., 2015). The addition of inflight proprioceptive countermeasures coupled with adequate resistance training could therefore help to mitigate the changes seen in response to prolonged exposure to microgravity (Bloomberg et al., 2015). The multifidus muscle is dense with muscle spindles (Nitz and Peck, 1986), and plays an important role in proprioception of the lumbo-pelvic region and control of the lumbar lordosis.

One disadvantage of the ARED is that it only involves movement in one dimension, but humans are designed to move in three dimensions. Elastic bands, such as Theraband, could be used to perform exercises in three dimensions. In addition, axial loading through the use of skinsuits is a possibility (Carvil et al., 2017). Different combinations of exercise countermeasures would be possible, for example, astronauts could perform exercises while wearing skinsuits, and while using technology based solutions already developed on Earth for conditions such as LBP. Virtual reality-based technology has successfully been used to administer LBP interventions (Park et al., 2013; Kim et al., 2014), where patients see themselves as a projected avatar. This could be used to monitor and correct posture, provide customized rehabilitation programs in order to strengthen muscles and increase endurance (Su et al., 2015). In addition, if astronauts were experiencing LBP on long duration missions and having difficulty with activation of the multifidus muscle, ultrasound imaging could be used to provide feedback and enhance MCT (Hides et al., 2012). Ultrasound imaging has been successfully used by crew on the ISS to provide examinations of the spine (Garcia et al., 2018), and ultrasound imaging could be a viable option for inclusion on long duration flights, due to the extremely compact nature of recently developed equipment.

## Conclusion

Exposure to microgravity is associated with LBP and an elevated risk of disc herniation on return to Earth. Understanding the mechanisms by which the exposure to microgravity affects the spine is important. This information is likely to guide in flight countermeasures. Understanding the effects of microgravity on the spine can provide new and potentially important information which could be used to design future interventions for people on Earth. As we move towards long-term missions, this reciprocal knowledge transfer could benefit both astronauts and people with chronic conditions such as LBP on Earth.

## Author Contributions

All authors listed have made a substantial, direct and intellectual contribution to the work, and approved it for publication.

### Conflict of Interest Statement

The authors declare that the research was conducted in the absence of any commercial or financial relationships that could be construed as a potential conflict of interest.
